# Cross-Protection Induced by Virus-like Particles Derived from the Influenza B Virus

**DOI:** 10.3390/biomedicines10071618

**Published:** 2022-07-06

**Authors:** Hae-Ji Kang, Ki-Back Chu, Keon-Woong Yoon, Gi-Deok Eom, Jie Mao, Fu-Shi Quan

**Affiliations:** 1 Department of Biomedical Science, Graduate School, Kyung Hee University, Seoul 02447, Korea; haedi1202@naver.com (H.-J.K.); kgang92@gmail.com (K.-W.Y.); ekd3910@naver.com (G.-D.E.); maojie@khu.ac.kr (J.M.); 2 Department of Medical Zoology, School of Medicine, Kyung Hee University, Seoul 02447, Korea; ckb421@gmail.com; 3 Medical Research Center for Bioreaction to Reactive Oxygen Species and Biomedical Science Institute, School of Medicine, Graduate School, Kyung Hee University, Seoul 02447, Korea

**Keywords:** influenza B virus, virus-like particle, vaccine, protection

## Abstract

The mismatch between the circulating influenza B virus (IBV) and the vaccine strain contributes to the rapid emergence of IBV infection cases throughout the globe, which necessitates the development of effective vaccines conferring broad protection. Here, we generated influenza B virus-like particle (VLP) vaccines expressing hemagglutinin, neuraminidase, or both antigens derived from the influenza B virus (B/Washington/02/2019 (B/Victoria lineage)-like virus, B/Phuket/3073/2013 (B/Yamagata lineage)-like virus. We found that irrespective of the derived antigen lineage, immunizing mice with the IBV VLPs significantly reduced lung viral loads, minimized bodyweight loss, and ensured 100% survival upon Victoria lineage virus B/Colorado/06/2017 challenge infection. These results were closely correlated with the vaccine-induced antibody responses and HI titer in sera, IgG, IgA antibody responses, CD4+ and CD8+ T cell responses, germinal center B cell responses, and inflammatory cytokine responses in the lungs. We conclude that hemagglutinin, neuraminidase, or both antigen-expressing VLPs derived from these influenza B viruses that were circulating during the 2020/21 season provide cross-protections against mismatched Victoria lineage virus (B/Colorado/06/2017) challenge infections.

## 1. Introduction

Influenza viruses are members of the family Orthomyxoviridae and are the causative agents of seasonal influenza. Influenza viruses A and B are of particular importance as their infection causes high mortality in humans [1,2]. The influenza B virus (IBV) has two antigenically distinct lineages, namely the B/Victoria and B/Yamagata which were determined by phylogenic studies in the 1970s [3,4]. IBV infection usually causes mild illness in healthy individuals, but it can cause serious illness in children and the elderly with a high complication rate [5,6]. As most of the focus was on the influenza A virus (IAV), the impact of IBV infections was largely undermined despite their significant contribution to the global influenza burden which was estimated to range from 290,000 to 650,000 annual deaths [7]. However, IBV-associated epidemics are beginning to emerge in the western hemisphere [8,9,10], possibly caused by the immune selection pressure exerted on the IBV hemagglutinin (HA) and neuraminidase (NA) antigens that consequently lead to the circulation of increasingly diverse IBV subclades [3]. The Center for Disease Control and Prevention (CDC), for example, reported 189 influenza-related deaths for the year 2019–2020 in the United States, and IBV accounted for 62% of the deaths [11].

Another factor contributing to emerging IBV cases throughout the globe is the lineage mismatch between circulating IBV and the vaccine. In the United States, it was reported that the correct circulating IBV lineage was included in the vaccine for only 5 out of the 10 influenza seasons from the years 2001 to 2011. Similarly, from 2003 to 2011 influenza seasons in Europe, lineage mismatch occurred in 4 out of the 8 consecutive seasons [12]. As indicated by limited antibody cross-reactivity between the 2 IBV lineages [13,14,15], vaccine-induced cross-protection is thought to occur at low levels. For this reason, the CDC currently recommends vaccination with a quadrivalent vaccine containing both lineages rather than a trivalent vaccine containing one B virus lineage [16]. Given the alarmingly increasing prevalence of IBVs and the lack of protective efficacy induced by lineage mismatch, developing an effective vaccine conferring broad protection for IBV has become a priority. To date, only a handful of IBV vaccine studies have been conducted using a wide array of vaccine platforms such as live-attenuated vaccines [17,18], vectored vaccines [19], inactivated influenza vaccine [20], or nanoparticles [21]. Nonetheless, improvements to the protective efficacy of these vaccines are needed. For example, live-attenuated vaccine immunization in children conferred 86% protection against the homotypic lineage, 55% against the homotypic lineage which underwent antigenic drift, and 31% against antigenically distant strains [22]. There is also the probability of reversion to the wildtype virulent phenotype, and, given the similar mutation rates for both influenza A and B viruses [23], additional safety precautions or alternative vaccine platforms should be used over live-attenuated vaccines.

One such alternative to the traditional influenza vaccine platform would be virus-like particles (VLPs). Evidently, the superior protective efficacy of VLP-based vaccines over inactivated vaccines has been widely reported. Trivalent VLP vaccines formulated using H1N1, H3N2, and influenza B viruses conferred protection exceeding those elicited by commercialized inactivated trivalent vaccines in mice and ferrets [24]. It has been previously demonstrated that immunization with the 1918 human influenza H1N1 VLPs conferred full protection against avian H5N1 virus challenge infection in ferrets, despite the significant virus mismatch between the two strains [25]. While their protective efficacy and breadth of protection against multiple IAVs have been extensively researched [26,27,28,29,30], there is a relative paucity of studies investigating such aspects against IBVs. Furthermore, as of the current moment, VLP-induced cross-protection between the two IBV lineages remains unreported. In this regard, developing a broadly protective VLP vaccine against IBVs could address the problem posed by virus mismatch and greatly contribute to a rational vaccine design strategy. Here, we generated six VLPs expressing HA, NA, or both derived from the 2020–2021 IBVs B/Phuket/3073/2013 (B/Yamagata/16/1980) and B/Washington/02/2019 (B/Victoria/2/1987). By using this immunogenic vaccine platform, we demonstrate that a high level of cross-protection between the two IBV lineages can be induced.

## 2. Materials and Methods

### 2.1. Mice, Cells, and Viruses

Female BALB/c mice (7-week-old, *n* = 8 per group) were purchased from Nara biotech (Seoul, Republic of Korea). All experimental procedures involving animals were performed following the Kyung Hee University Institutional Animal Care and Use Committee guidelines (approval number: KHSASP-20-604). *Spodoptera frugiperda* 9 (*Sf*9) cells were used to generate recombinant baculovirus (rBV) and VLPs. Sf9 cell suspension cultures were maintained in serum-free SF900II medium (Invitrogen, Carlsbad, CA, USA) at 27 °C in spinner flasks at 135 rpm. Madin–Darby canine kidney (MDCK) cells were maintained in Dulbecco’s modified Eagle’s medium (DMEM) with 10% fetal bovine serum (FBS) and used for virus plaque assays. MDCK-adapted influenza B/Colorado/06/2017 (Victoria/2/87 lineage) virus was prepared by inoculating the viruses at a multiplicity of infection (MOI) of 0.02. After serial passaging, adapted viruses were collected and stored at −80 °C until use.

### 2.2. Generation of Recombinant Baculovirus and Virus-like Particles

VLPs were produced following the method previously described [31]. The pFastBac plasmids containing codon-optimized hemagglutinin (HA) and neuraminidase (NA) genes of B/Washington/02/2019 (B/Victoria/2/1987) and B/Phuket/3073/2013 (B/Yamagata/16/1988) were purchased from GenScript (Piscataway, NJ, USA). The hemagglutinin and neuraminidase proteins derived from B/Washington/02/2019 and B/Phuket/3073/2013 were abbreviated as Hy, Ny, Hv, and Nv, respectively. Recombinant plasmids containing Hv, Nv, Hy, or Ny were transformed into DH10Bac competent cells and bacmid DNA from successful clones was acquired. Bacmid DNA derived from Hv, Nv, Hy, or Ny was transfected into Sf9 cells using Lipofectamine 2000 (Invitrogen, Carlsbad, CA, USA) and recombinant baculoviruses (rBVs) were produced. VLPs were produced by co-infecting rBVs expressing the H and N proteins of influenza B virus along with the influenza A virus M1-expressing rBVs at 0.2 MOI. This combinatorial approach was used to generate 6 different VLPs as follows: Hv VLPs (Hv+M1), Nv VLPs (Nv+M1), HvNv VLPs (Hv+Nv+M1), Hy VLPs (Hy+M1), Ny VLPs (Ny+M1), and HyNy VLPs (Hy+Ny+M1). Sf9 cell culture supernatants containing the VLP fraction were harvested at 3 days post-infection (dpi). Cells were sedimented by centrifuging at 6000 rpm, 30 min, 4 °C. The supernatant fraction was centrifuged at 30,000 rpm, 20 min, 4 °C. Pelleted VLPs were purified through a 15%–30%–60% sucrose gradient and stored at −80 °C until use.

### 2.3. Western Blot

Six VLPs (Hv, Nv, HvNv, Hy, Ny, and HyNy) were loaded and separated on a 10% SDS-PAGE gel. After transferring the proteins to nitrocellulose membranes at 70 V for 90 min, membranes were blocked for 30 min using 5% skim milk prepared in TBS with 0.1% Tween 20. Mouse anti-M1 monoclonal antibody was purchased from Abcam (Cat# 22396, Cambridge, UK) and rabbit anti-NA monoclonal antibody (HCA-2) was kindly provided by Dr. Sang-moo Kang, Georgia State University, GA, USA [32]. Sera of influenza B/Colorado/06/2017-infected mice (1:500), anti-NA monoclonal antibody (1:10,000), and anti-M1 monoclonal antibody (1:3000) were diluted in TBST and used to probe the membranes overnight at 4 °C. The next day, membranes were repeatedly washed with TBST and incubated with either horseradish peroxidase (HRP)-conjugated anti-mouse or anti-rabbit IgG secondary antibodies (1:2000 and 1:10,000 dilutions in TBST, respectively). Protein expressions were detected following enhanced chemiluminescence exposure and images were acquired on ChemiDoc Imaging System (Bio-Rad, Hercules, CA, USA).

### 2.4. Hemagglutinin Activity

HA activity was measured using round-bottom 96-well microtiter plates following the method previously described [31]. Briefly, 1 μg VLP samples were added and serially diluted with PBS. Next, 50 μL of 0.5% chicken red blood cell suspensions were inoculated into each well, and plates were incubated for 2 h at RT.

### 2.5. Neuraminidase Activity

Neuraminidase activity of the VLPs was measured using the Amplex Red Neuraminidase Assay Kit (Invitrogen, MA, USA) following the manufacturer’s instructions. In brief, 10 μg of VLP samples were diluted with 50 μL of Amplex Red reaction buffer and then added to a flat-bottom 96-well plate. Amplex red working reagent was prepared and 50 μL of this solution was added to each well. Plates were incubated in the dark for 30 min at 37 °C and absorbance readings at 570 nm were measured.

### 2.6. Transmission Electron Microscopy (TEM)

TEM (JEM-1400 Plus at 120 kV and JEM-1000BEF at 1000 kV, JEOL Ltd., Tokyo, Japan) was used to observe the morphology of the influenza B VLPs. VLPs samples were placed on formvar-amorphous carbon-coated copper grid (Ted Pella, Redding, CA, USA) and stained with 2% uranyl acetate. Stained grids were observed under a TEM and images were captured to confirm VLPs morphology.

### 2.7. Hemagglutinin Inhibition Assay

Influenza B/Colorado/06/2017 virus was used as HI antigens to test all immunized mice serum samples in this study. Serum samples underwent a standard receptor destroying enzyme (RDE) treatment at 37 °C overnight followed by inactivation at 56 °C for 30 min. Initially, 25 μL of PBS was added to each well of the U-bottom 96-well plates. Next, 50 μL of RDE-treated serum samples at 1:10 dilution was dispensed into the first well prior to 2-fold serial dilution. After dispensing 25 μL of pre-titrated viruses (8 HA units/50 μL), plates were incubated at room temperature for 30 min to allow antibody-virus neutralization. Finally, 0.5% chicken RBCs were added to each well and HI titers were determined.

### 2.8. Antibody Secreting Cells (ASC) Assay

ASC assays were performed as previously described [31]. Inactivated influenza B virus (B/Colorado/06/2017) antigen was dissolved in carbonate coating buffer at a concentration of 4 μg/mL and used to coat 96-well microtiter plates overnight at 4 °C. Plates were blocked with 0.2% gelatin, and lung cells (1 × 10^6^ cells/well) resuspended in complete RPMI media were inoculated into each well. Plates were incubated for 5 days at 37 °C with 5% CO_2_. After removing culture supernatants, HRP-conjugated anti-mouse IgG or IgA antibodies were added to each well and optical density values at 450 nm were measured.

### 2.9. Antibody Secreting Cells (ASC) Assay

Mice (*n* = 8 per group) were intramuscularly immunized twice with 10 μg of Hv VLPs, Nv VLPs, HvNv VLPs, Hy VLPs, Ny VLPs, or HyNy VLPs in 100 μL of PBS at a 4-week intervals. Four weeks after each immunization, blood samples were collected from each mouse via retro-orbital plexus puncture. Four weeks after the final immunization, immunized mice were challenged with a lethal dose of B/Colorado/06/2017 (5LD50) and the protective efficacy of the VLPs was evaluated.

### 2.10. Enzyme-Linked Immunosorbent Assay (ELISA) for Antibody and Cytokine Production

Influenza virus-specific IgG and IgA antibody responses were determined from sera and lung homogenates by ELISA as described previously [31]. Inactivated influenza virus (B/Colorado/06/2017) at a concentration of 4 μg/mL was used to coat the 96-well microtiter plates. After coating the plates overnight at 4 °C, plates were blocked with 0.2% gelatin and incubated with either lung homogenates or sera. HRP-conjugated secondary antibodies were added to each well and OD 450 nm readings were measured. Concentrations of the pro-inflammatory cytokines interferon-gamma (IFN-γ), interleukine-6 (IL-6), and tumor necrosis factor-α (TNF-α) in lung extracts were measured using the BD OptEIA ELISA kits following the manufacturer’s instructions as described previously [31,33].

### 2.11. Flow Cytometry Assessment of Lung Cell Populations

To analyze the VLP vaccine-induced changes in immune cell populations, flow cytometry was performed. Briefly, lung tissues were harvested after 4 dpi with the influenza B virus challenge infection. After homogenizing the tissues, single cell populations of lung cells were prepared by Percoll density gradient as described previously [31]. Cells were stimulated with inactivated B/Colorado/06/2017 virus antigen (0.4 ug/mL) for 2 h at 37 °C. CD4+ T cell, CD8+ T cell, B cell, and germinal center (GC) B cell populations were measured by staining the cells with fluorophore-conjugated CD3, CD4, CD8, B220, GL7, CD19, and IgD antibodies (BD Biosciences, San Diego, CA, USA). Stained cells were acquired on C6 flow cytometer and analyzed using C6 Analysis Software (BD Bioscience, San Diego, CA, USA).

### 2.12. Lung Virus Titer

Lung virus titers at 4 dpi were determined by plaque assay as previously described [34]. MDCK cells were seeded in a 12-well culture plate and grown until confluent. Lung homogenates from mice were serially diluted in serum-free DMEM media and inoculated into respective wells. After incubating the plates at 37 °C for 1 h, diluted homogenates were aspirated and cells were gently overlayed with an overlay medium consisting DEAE dextran, non-essential amino acids, glutamine, and trypsin. Plates were incubated for 3 days at 37 °C with 5% CO_2_. Cells were fixed with 0.25% glutaraldehyde and stained with 1% crystal violet solution for plaque counting.

### 2.13. Statistical Analysis

GraphPad Prism 8 software (GraphPad Software, Inc., San Diego, CA, USA) was used to perform statistical analysis. Statistical significance between the means of each group was evaluated using one-way analysis of variance (ANOVA) with Tukey’s post hoc tests. Data sets were presented as mean ± SD and asterisks were used to denote statistical significance. Differences were considered statistically significant at * *p* < 0.05, ** *p* < 0.01, *** *p* < 0.001.

## 3. Results

### 3.1. Characterization of the Influenza B VLPs Containing HA and NA

**Figure 1:**
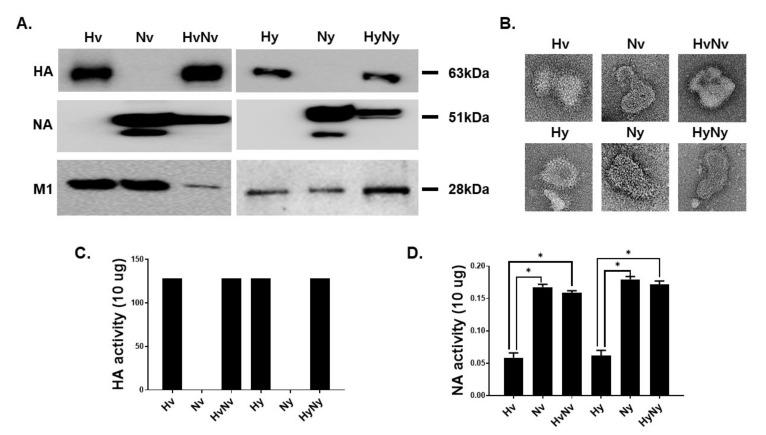
Characterization of the influenza B VLP vaccines. Western blots were performed and the expressions of HA, NA, and M1 proteins were confirmed using polyclonal and monoclonal antibodies (**A**). VLPs were negatively stained and visualized under the transmission electron microscope (**B**). Chicken RBCs were used to assess the HA activity of the VLPs (**C**), and NA activity was evaluated using the Amplex Red assay kit (**D**). Data are expressed as mean ± SD with asterisks denoting statistical significance between groups (* *p* < 0.05).

VLPs expressing Hv, Nv, Hy, and Ny proteins from the two lineages were generated. To confirm successful expression of the proteins of interest, VLPs were analyzed by western blot. Expressions of Hv and Hy antigens were observed at 63 kDa when membranes were probed with the sera of B/Colorado/06/2017-infected mice, whereas antigen-antibody interactions were not detected from the neuraminidase-expressing VLPs. As expected, NA-specific antibody only interacted with the Nv and Ny VLPs with a molecular weight of 51 kDa. HA and NA antigens were detected from VLPs co-expressing both antigens. Similarly, successful expression of M1 core antigen was detected from all VLP samples (Figure 1A). Morphological characteristics of VLPs were evaluated using TEM. Protrusion of antigen spikes through the lipid envelope was detected from each VLPs (Figure 1B). HA activity of each VLPs was determined by measuring the titer at which chicken RBC agglutination occurred. HA activities were measured from HA-incorporated VLPs, all of which showed 128 HA titers. As expected, HA activities were not detected from Nv and Ny VLPs (Figure 1C). Similar to the HA activity, NA activities were only detected from VLPs expressing the neuraminidase antigen as Hv and Hy NA activities were significantly less than those of NA-expressing VLPs (Figure 1D).

### 3.2. Immunization with Influenza B VLPs Induced Antibody Response in Serum

**Figure 2:**
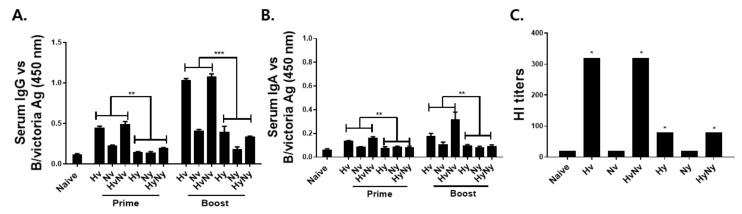
Antibody responses and hemagglutinin inhibition in sera. BALB/c mice (*n* = 8 per group) were intramuscularly immunized with VLPs vaccines and sera were collected at regular intervals after each immunization. B/Colorado/06/2017 virus-specific IgG (**A**) and IgA (**B**) antibody responses, as well as HI titers (**C**), were determined. Data are expressed as mean ± SD with asterisks denoting statistical significance between groups (* *p* < 0.05, ** *p* < 0.01, *** *p* < 0.001).

To validate the immunogenicity of influenza B VLPs, mice were intramuscularly immunized with 10 ug of Hv, Nv, HvNv, Hy, Ny, or HyNy VLPs using a prime-boost regimen and sera were collected 4 weeks after each immunization. B/Colorado/06/2017-specific IgG responses were detected 4 weeks post-prime immunization, but these were only elicited from Hv and HvNv VLPs. Marginal increase in IgG response was detected from Nv VLPs, whereas the antibody responses were negligibly changed in the Hy, Ny, and HyNy VLPs (Figure 2A). Upon boost immunization, a drastic increase in virus-specific IgG responses from all VLPs, except for Ny VLPs. At both time points, Hv and HvNv VLP-induced antibody responses were significantly greater than those of Hy or HyNy VLPs. IgA antibody responses against the B/Colorado/06/2017 virus were strikingly similar to IgG, albeit at lower levels. Even after prime and boost immunizations, Hy, Ny, and HyNy VLP-induced antibody responses were negligibly changed (Figure 2B). Significantly higher IgA responses were elicited by the Victoria lineage VLPs following prime and boost immunizations. The lineage of each antigen expressed in the VLPs strongly influenced antibody induction in mice. Resultantly, homotypic challenge infection with a Victoria lineage influenza B virus led to a higher antibody response being induced in the Hv, Nv, and HvNv VLP-immunized group. HI titers are correlated with the protective efficacy of influenza antibody responses. To confirm the efficacy of the Hv, Nv, HvNv, Hy, Ny, or HyNy VLPs, we examined their HI titers (Figure 2C). The HI antibody titers against the B/Colorado/06/2017 virus elicited by the Hv and HvNv VLPs were 4-fold higher than those elicited by the Hy and HyNy VLPs.

### 3.3. Immunization with Influenza B VLPs Induced Antibody Responses in the Lung

**Figure 3:**
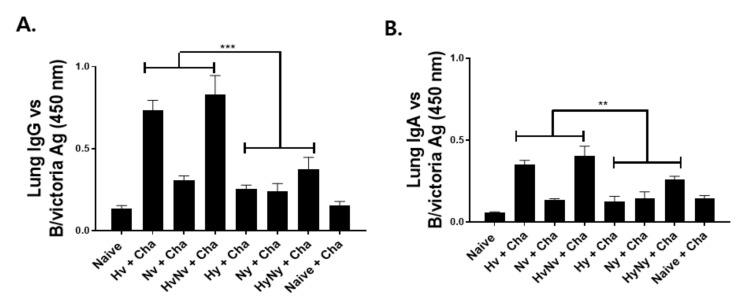
Antibody responses in the lung. Immunized mice were intranasally infected with a lethal dose of B/Colorado/06/2017 at 4 weeks after boost immunization. Lungs from individual mice were collected at 4 dpi to evaluate pulmonary IgG (**A**) and IgA (**B**) antibody responses by ELISA. Naïve+Cha refers to those naïve mice who were challenge infected with a lethal dose of influenza virus. Data are expressed as mean ± SD with asterisks denoting statistical significance between groups (** *p* < 0.01, *** *p* < 0.001).

To determine the effects of influenza B VLP immunization, groups of mice were intramuscularly immunized and lung antibody responses specific to B/Colorado/06/2017 influenza B virus were evaluated. Consistent with the serum antibody responses, stronger pulmonary antibody responses were induced in Victoria lineage VLPs than in Yamagata lineage VLPs. Antigen-specific IgG responses from Hv and HvNV, in particular, were significantly higher than those of Hy or HyNy (Figure 3A). A similar trend was observed for the lung IgA, as Hv and HvNv responses were substantially greater than Hy or HyNy VLP-induced responses (Figure 2B).

### 3.4. Influenza B VLPs Immunization Induces Effective ASC Responses

**Figure 4:**
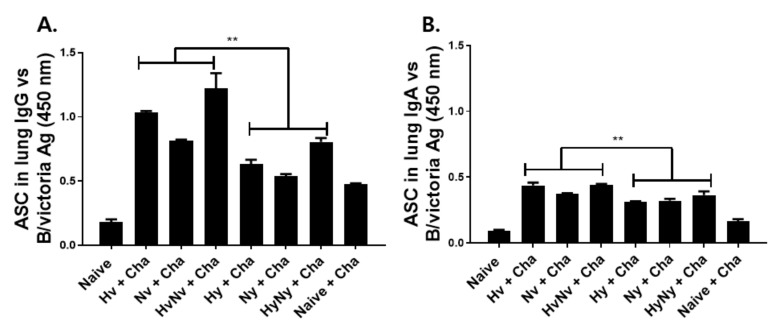
Antibody-secreting cell (ASC) responses in the lung. Immunized mice were challenge-infected with B/Colorado/06/2017 virus and at 4 dpi, lung cells were collected to measure ASC responses. ASC IgG (**A**) and IgA (**B**) antibody responses were evaluated by ELISA. Naïve+Cha refers to those naïve mice who were challenge infected with a lethal dose of influenza virus. Data are expressed as mean ± SD with asterisks denoting statistical significance between groups (** *p* < 0.01).

To further investigate the B cell function induced by VLP immunization, murine cells were acquired after 4 dpi and cultured for 5 days to analyze the ASC responses. VLP immunization in mice, regardless of the virus lineage, induced IgG and IgA ASC responses against the B/Victoria antigen. All VLPs induced higher levels of ASC responses compared to Naïve and Naïve+Cha groups. For IgG ASC, HA-expressing VLPs elicited higher ASC responses than those of NA VLPs. This was a common feature observed in both VLP lineages (Figure 4A). While Hy and HyNy VLP immunization induced ASC responses to an extent, these levels were significantly greater in those of Hv and HvNv VLP groups. A strikingly similar pattern was observed from IgA ASC (Figure 4B). As with IgG ASC, VLP immunization induced substantially higher IgA ASC responses compared to the control groups.

### 3.5. Influenza B VLPs Induced a Higher Level of Cellular Immune Cell Response

**Figure 5:**
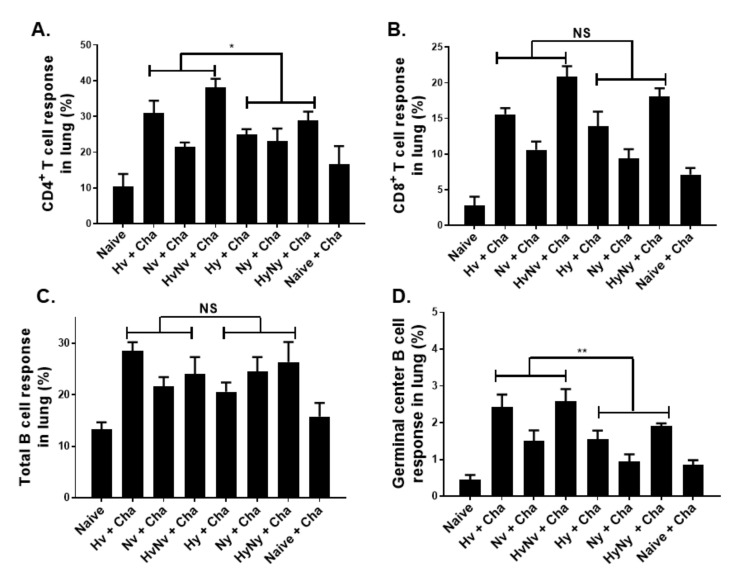
Cellular immune responses after influenza B virus challenge infection. Lung cells were stained with fluorophore-conjugated antibodies and gated to quantify changes in various cell populations. CD4+ T cell (**A**), CD8+ T cell (**B**), total B cell (**C**), and germinal center B cells (**D**) inductions post-challenge with B/Colorado/06/2017 virus were evaluated by flow cytometry. Naïve+Cha refers to those naïve mice who were challenge infected with a lethal dose of influenza virus. Data are expressed as mean ± SD with asterisks denoting statistical significance between groups (* *p* < 0.05, ** *p* < 0.01; NS: not significant).

Cellular immune response inductions in the lungs post-challenge infection were assessed by flow cytometry. CD4+ T cell proliferation was observed in VLP-immunized mice. VLPs expressing both HA and NA antigens elicited a stronger immune response than those expressing either HA or NA alone (Figure 5A). Despite the challenge infection with B/Victoria lineage virus, Yamagata lineage VLPs still induced T cell proliferation in mice, albeit at significantly lesser levels than Victoria lineage VLPs. While differences in CD4+ T cell responses were observed, such findings were not detected for CD8+ T cells as their overall inductions were comparable for both lineages (Figure 5B). Similarly, while total B cells were induced to a certain extent, significant differences between the groups were not observed (Figure 5C). GC B cells were induced to an extent and significant differences between the lineages were observed. Expectedly, VLPs expressing the HA antigen derived from Victoria lineage induced stronger GC B cell responses than those of Yamagata lineage (Figure 5D). Overall, the percentage of activated CD4+ T cell, CD8+ T cell, total B cell, and GC B cell were significantly increased in all immunization groups compared to naïve and naïve+cha controls.

### 3.6. Influenza B VLPs Immunization Inhibits Lung Inflammatory Cytokine Expressions

**Figure 6:**
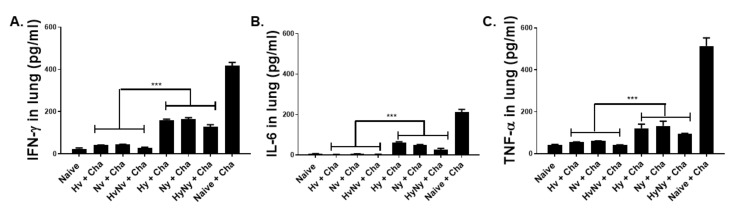
Pro-inflammatory responses in the lung after influenza B virus challenge infection. Levels of inflammatory cytokines IFN-γ (**A**), IL-6 (**B**), and TNF-α (**C**) were determined in the lung extracts at 4 dpi with B/Colorado/06/2017 virus. Naïve+Cha refers to those naïve mice who were challenge infected with a lethal dose of iInfluenza virus. Data are expressed as mean ± SD with asterisks denoting statistical significance between groups (*** *p* < 0.001).

Drastic reductions in pro-inflammatory cytokine expressions were detected in all immunization groups. IFN-γ expression in mice immunized with the B/Victoria virus-derived antigens was near basal levels, reflecting those of naïve control (Figure 6A). While substantial reductions compared to Naïve+Cha control were also observed from Yamagata lineage VLPs, the differences between the two lineages were significantly different. This was also observed for IL-6, as their production from Victoria lineage VLP-immunized mice was strikingly lower than those of Yamagata lineage VLP groups (Figure 6B). Consistent with this trend, TNF-α expressions also demonstrated this phenomenon. While Hy, Ny, and HyNy VLPs all lessened the production of TNF-α in mice, these were inhibited to a much greater extent in the Hv, Nv, and HvNv VLP groups (Figure 6C). Overall, the production of these cytokines in the Victoria lineage VLP groups resembled those of the naïve control, and differences were negligible.

### 3.7. Influenza B VLP Immunization Contributes to Protecting Mice from Lethal Influenza B Virus Challenge

**Figure 7:**
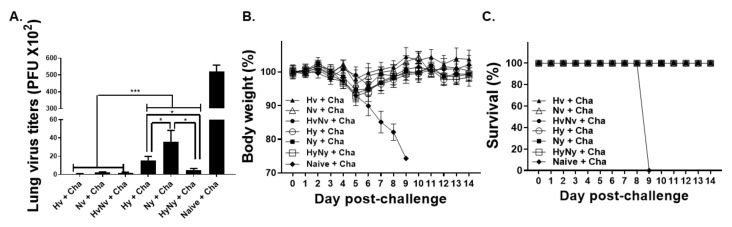
Lung virus loads, body weight changes, and survival after lethal influenza B virus challenge. BALB/c mice were challenged with a lethal dose of influenza B virus and vaccine efficacy was evaluated by measuring the lung virus titer (**A**), bodyweight changes (**B**), and survival (**C**). Naïve+Cha refers to those naïve mice who were challenge infected with a lethal dose of influenza virus. Data are expressed as mean ± SD with asterisks denoting statistical significance between groups (* *p* < 0.05, *** *p* < 0.001).

To confirm the protective efficacy of the influenza B VLPs, mice were challenge infected with a lethal dose of B/Colorado/06/2017 virus and changes to lung virus titer, bodyweight loss, and survival were assessed. VLPs, irrespective of the antigen lineage, protected mice against the challenge infection. Compared to the naïve+cha control, significant lung virus titer was observed in all immunization groups (Figure 7A). Drastically reduced lung virus titers were observed from the Victoria lineage VLPs, whose titers were significantly lower than those of Yamagata lineage VLPs. Notably, HyNy VLP-induced lung titers were significantly lower than either Yamagata lineage HA or NA-expressing VLPs. Furthermore, HyNy lung titers were comparable to those elicited by Victoria lineage VLPs, thus signifying the presence of cross-lineage protection. Vaccine-induced protection was further evaluated by measuring bodyweight reduction and survival for 2 weeks. All of the mice belonging to the naïve+cha group perished by 9 dpi, with noticeable bodyweight reduction occurring from 3 dpi onwards (Figure 7B). Contrary to this, the bodyweights of Hv, Nv, HvNv, Hy, Ny, and HyNy dropped 2.2%, 3.7%, 1%, 6.5%, 7.2%, and 5.4%, each respectively by 5 dpi. This was immediately followed by gradual bodyweight recovery and mice were fully recovered around 9 dpi. None of the immunized mice perished, showing 100% survival rate, whereas all of the mice in naïve+cha died (Figure 7C). These results indicated that VLP vaccines derived from the two IBV lineages of the 2020/21 influenza season protected mice from mismatched B/Colorado/06/2017 virus infections.

## 4. Discussion

In the present study, VLPs expressing the HA and NA antigens from two different lineages of influenza B virus were generated and their protective efficacies were evaluated in mice. Our results demonstrated that VLPs expressing the HA or NA antigens of influenza B viruses can elicit both cellular and humoral immune responses to confer protection in mice. Immunizing mice with the VLPs significantly lessened the pro-inflammatory cytokine responses. Furthermore, cross-immunity resulting in homotypic and heterotypic protection was confirmed when mice were challenge-infected with a lethal dose of B/Colorado/06/2017 virus. Importantly, none of the immunized mice perished and bodyweight changes were generally negligible.

Antibody production is one of the most important functions of the immune response and is the basis for the vast majority of successful vaccination strategies [35,36]. GC B cells and ASCs, in particular, are crucial for sustaining high levels of antigen-specific serum antibody responses [37,38]. In line with this notion, VLP-immunization induced sufficiently high levels of antibody levels which correlated with significant increases in GC B cell populations and ASC responses. Based on the serum antibody responses induced by Yamagata lineage VLPs, we initially speculated that heterotypic protection would not occur or only occur to a small extent. Cross-reaction between the two lineages was observed as we initially anticipated. Evidently, several studies involving human subjects have reported antibody cross-reaction between the two opposing lineages of IBV. It was reported that primary infection with either lineage of influenza B virus elicits antibody-dependent cell cytotoxicity-mediating antibodies that can detect the HA stalks of opposing lineage [39]. Another study reported the successful cross-reaction of B/Yamagata lineage HA antigen with the antibodies elicited upon immunization with the B/Victoria HA-expressing trivalent vaccine [40]. Cross-reactive antibody response in IBV was also observed from secretory IgA [41]. Consistent with these reports, our results also confirmed that mice immunized with the Yamagata lineage HA-expressing VLPs also induced considerable IgG levels against B/Victoria antigen, while they occurred to a much weaker extent in B/Yamagata NA-expressing VLPs.

Expectedly, irrespective of IBV lineage, VLPs displaying the HA antigens on the surface elicited stronger virus-specific serum antibody responses than NA VLPs. This phenomenon can be explained by the quantitative differences in viral HA and NA antigen expressions. NA antigen expression on the viral surface tends to be substantially lower than the HA antigen, with their relative ratio being 1:4 or [42]. Since the surface of the inactivated virus used as coating antigen is heavily populated with HA antigens, serum antibody interaction with the NA antigen is likely to occur to a far lesser extent. Such intravirionic antigenic competition is thought to be responsible for the predominance of antibodies targeting the HA antigen. Previous findings have reported that influenza B VLPs are capable of eliciting HA and NA activities. For instance, mammalian cell-produced influenza B VLPs expressing the surface proteins of B/Phuket/3073/2013 virus had HI titers ranging from 197 to 260 [43]. This was surprisingly close to the HI titer of 128 reported from our study, despite using a different dose and different expression system for VLP production. While the aforementioned study also measured the NI titers, direct comparison with our results is difficult as we expressed our data using absorbance readings.

Studies investigating the immunological parameters associated with protection against IBV infections are severely limited. CD4+ and CD8+ T cells are important effectors for regulating IBV infection. Earlier study has revealed that mice could survive IBV challenge infection even upon antibody and B cell depletion, due to the presence of CD4+ and CD8+ T cells. However, ablating either of the two or both T cell subsets resulted in complete abrogation of protection [44]. Furthermore, CD8+ T cells specific to one IBV lineage can cross-react with the opposing lineage [14]. Based on these previous reports, we anticipated that homotypic and heterosubtypic protection would occur to an extent as significantly high levels of CD4+ and CD8+ T cell responses were elicited. In our study, 10-fold or more virus titer reductions were observed in all immunization groups. Recently, it was reported that CD4+ T cells become highly localized in the lung tissues post-infection with IBV [45]. Partial enrichment of CD4+ T cells in the lungs was observed in our study, thus confirming the findings of the previous work. Overall, these cellular and humoral aspects of the immune responses most likely contributed to conferring strong cross-protection against opposing IBV lineage.

Nanoparticles expressing the HA stem, NA active sites, and M2 ectodomain epitopes were also reported to confer varying degree of protection against IBVs (B/Florida/04/2006), which were strongly influenced by antigen epitope formulations [21]. In the present study, VLP-based vaccines ensured that all of the immunized mice survived regardless of lineage, and this was observed from VLPs expressing only a single type of antigen. The aforementioned study also reported that co-formulated vaccines expressing multiple epitopes enhance protection, which was also observed in our study. In another study, baculoviruses were used to express recombinant surface antigens of IBVs and their protective efficacies were compared to both conventional influenza vaccines and live influenza vaccines. While all of the vaccines expressing HA antigen conferred protection against homotypic challenge in mice, protection against distant IBV variants was only observed from vaccines that co-expressed immunogenic amounts of NA antigens which were observed from neither conventional nor live influenza vaccines [46]. Consistent with this finding, the HyNy VLPs used in the present study conferred protection against B/Victoria strain, which were better than VLPs expressing either Hy or Ny alone.

Given the nature of baculovirus expression system, high immunogenicity and the protection resulting from VLP immunization were as expected. One unique feature of these non-pathogenic baculoviruses that contributed to these findings is their adjuvant property. Co-presence of baculoviruses along with baculovirus-derived VLPs, even at trace amounts, can function as potent adjuvants to promote both humoral and CD8+ T cell responses [47]. Interestingly, immunizing mice with the recombinant adenovirus expressing the nucleoprotein of IBV conferred protection when administered through the intranasal route but not intramuscular route [19]. In another study, intranasally immunizing guinea pigs with IBV NA antigen reduced the lung virus titers but such findings were not observed when the antigens were intramuscularly administered [48]. In stark contrast to these studies, we found that intramuscular administration of VLPs reduces virus titer and confers protection in mice. It is important to note that when it comes to broad protection, IN influenza VLP administration confers greater protection than IM immunization [25]. Given that IM immunization with IBV VLPs conferred broad protection in our study, we think that evaluating these VLPs using IN route would drastically strengthen the protective efficacy of the VLP vaccines. Considering the ease of administration, intramuscular VLP immunization could serve as an alternative to these methods. A recombinant adenovirus vaccine expressing the synthetic HA2 antigen was shown to be effective against influenza B virus infection, as the vaccine enhanced the live GC B cell percentages in the draining lymph nodes and reduced the lung viral loads in mice [49]. Although GC B cell populations were assessed in a different organ in our study, notably the lung as it is the primary infection site, the results were similar as significantly incremental changes in GC B cell populations were observed. With regards to lung viral titers, VLP-induced virus inhibition was substantially greater than the recombinant viral vaccine. In conclusion, we generated VLP vaccines that provide homotypic and heterotypic protection against IBV infection in mice. Our findings offer promising prospects for future influenza B virus vaccine development.

## Data Availability

Data supporting the findings of this study are contained with the article.
